# Outlining the role of experiential expertise in professional work in health care service co-production

**DOI:** 10.1080/17482631.2021.1954744

**Published:** 2021-07-25

**Authors:** Hannele Palukka, Arja Haapakorpi, Petra Auvinen, Jaana Parviainen

**Affiliations:** a R&D and Innovation Services, Tampere University of Applied Sciences, Tampere, Finland; b Work Research Centre (WRC), Tampere University, Tampere, Finland; c The Faculty of Social Sciences, Tampere University, Tampere, Finland

**Keywords:** Patient and public involvement, experts by experience, opiate substitution treatment, social representation theory, social identity

## Abstract

Patient and public involvement is widely thought to be important in the improvement of health care delivery and in health equity.**Purpose:** The article examines the role of experiential knowledge in service co-production in order to develop opiate substitution treatment services (OST) for high-risk opioid users.**Method:** Drawing on social representations theory and the concept of social identity, we explore how experts’ by experience and registered nurses’ understandings of OST contain discourses about the social representations, identity, and citizenship of the participants and the effects these may have on developing or hindering inclusive and bottom-up forms of patient and public involvement.**Results:** The meeting sessions that potentially offer room for creativity and problem-solving fail to provide any new propositions for fixing the system. The health care professionals primarily identify themselves as regulators who protect the correctness of their actions and show little interest in considering experiential knowledge on opioid addiction. **Conclusion:** The participation of patients has been one of the prominent reforms implemented in health care. The goal of client-centered thinking is often emphasised; however, the implementation is not simple due to the strongly institutionalised knowledge and related working patterns and practices in health care.

## 1. Introduction

### 1.1. Experts by experience in the co-production of health care services

Patient and public involvement (PPI) is widely thought to be important in the improvement of health care delivery and in health equity (World Health Organization [WHO], 2008; World Bank, 2006). There has been exponential growth in the employment of experts by experience in the health and social care sectors in the UK, and recently this trend has spread to the Nordic countries (Cleary et al., 2018). In Finland, the citizen’s role as a service provider is growing because the current policy demands their active engagement in the co-production of health care services. The objectives directed towards engaging the citizens are written into the Finnish policy programmes and strategies, which emphasizes the importance of experiential expertise (Palukka et al., 2019).

Participation has increasingly become a means and an end for successful and “empowering” Finnish social policy (Meriluoto, 2018). The term *experts by experience* is used to refer to former service users or social welfare clients who participate in various roles by drawing on their experiential knowledge.^1^ Their activities vary from being consultants and evaluators in service co-production to being lecturers, spokespeople, and peer-supporters. Experts by experience are recognized actors in health services because they provide a variety of perspectives and feedback that can be used to develop social and health services.

The growing popularity of experiential expertise in social and health services is explained by the paradigm shift in mental health and substance abuse work, which has shifted from the institutional paradigm to the rehabilitation paradigm since the 1960s in Finland. The institutional paradigm is associated with the idea of a patient as a passive subject, whereas, from the perspective of the rehabilitation paradigm, the patient is seen as an active participant (Aspvik, 2003). As the service system becomes more focused on outpatient care, the responsibility of peers and relatives for the rehabilitation of both those with mental health problems and substance abusers has increased (Nyman & Stengard, 2001; Wahlbeck, 2007).

Although society utilizes experts by experience in many different ways, it is nevertheless a personal process in which the role of the expert by experience is contradictory, and constantly changing. An expert by experience can be on an equal footing with a professional, and professionals can appreciate their expertise. On the other hand, their experience-based knowledge is not always recognized, and their expertise is rarely taken into account (Palukka et al., 2019). By applying Miranda Fricker’s (2007) conceptualization of testimonial injustice, we suggest that former opioid abusers suffer easily a credibility deficit due to harmful social stereotypes. Their testimony is not believed, taken seriously, or considered interesting or relevant (Auvinen et al., 2021).

Expertise by experience as a concept and a practice has been traced back to the “third way” health and social care reforms in Europe, which sought to craft a new, active role for the service user (Barnes & Cotterell, 2012a; Fox et al., 2005; Tehseen, 2013; Wilson, 2001). Similarly to many other participatory measures, these service user-involvement initiatives were introduced as a response to an array of problems—both social and economic (Barnes & Cotterell, 2012b; Lewis, 2010, pp. 277–278; Newman & Clarke, 2009, pp. 134–139; Stewart, 2013).

The ideas and developments that have come from service users and their movements have been based on their particular knowledge. As has been said, what distinguishes service-user knowledge and what is unique about it is that it is based on direct experience. Unlike other stakeholders in the policy process—for example, policymakers, managers, practitioners, researchers, or educators—what distinguishes their perspective is that it rests on their role as the end users of policy and practice. They “know” through lived experience (Beresford, 2010).

This is the basis and starting point for their views, ideas, and proposals. Only they can truly be said to “know” what policy and provision are like, rather than what its aims, intentions, and rationale are, since they live them. As Alison Faulkner and Phil Thomas have suggested, for example, for mental health service users, the voice of experience is breaking through longstanding barriers of enforced silence, incarceration, and compulsion (Faulkner & Thomas, 2002).

However, in service user-involvement schemes, the service users’ knowledge is most often referred to as “secondary” or “alternative” knowledge, hence implying that they serve a complementary role. Their expertise is often defined being of a practical nature, adding something valuable, but not fundamental, to the discussion. “First knowledge” is situated elsewhere, allowing the secondary knowledge to be evaluated vis-à-vis it (Barnes & Cotterell, 2012a). Furthermore, it has been noted that the participants are often invited to take part based on their experience-based knowledge, but are required to transcend their personal views when actually engaging in the activities of participatory governance in order for their participation to be considered legitimate (see, e.g., Lehoux et al., 2012; Neveu, 2011, p. 151; Thévenot, 2007, p. 420).

### 1.2. The role of experts by experience as the consultants and evaluators in service co-production in order to develop opiate substitution

Our interest was in finding out the role of experts by experience as consultants and evaluators in service co-production in order to develop opiate substitution treatment (OST) services. The chronic disease of opioid use disorder is increasingly a global health issue. In the European Union (EU), for instance, in 2018 there were about 1.3 million high-risk opioid users (mainly heroin users) aged 15–64. In Finland, there were about 15,000 high-risk opioid users (0.3% of the Finnish population). Out of this group, 3329 persons received OST in 2018 (EMCDDA, 2019).

OST is a medically supervised treatment for opioid-dependent persons using substitution drugs like methadone and buprenorphine. These are drugs that have a similar action to the drug of dependence, thereby alleviating withdrawal symptoms and suppressing the craving for illicit opiates.

A prerequisite for OST is that the patient commits to abstaining from the drug of dependence which is monitored regularly during treatment. The monitoring is carried out via a urine sample, taken under the supervision of a qualified health care professional (Mykkänen et al., 2015). When starting OST, the rehabilitated person becomes under severe control and, at the same time, loses her freedom.

This article examines the role of experiential knowledge in service co-production in order to develop OST services for high-risk opioid users. The article seeks to present answers to the following questions: (1) In what ways do the experts by experience attempt to legitimize their expertise that is based on experience in relation to the expertise of health care professionals that is based on professional knowledge? (2) In what ways do the registered nurses justify their professional expertise in relation to the experiential knowledge of experts by experience?

## 2. Research methodological design

Drawing on social representation theory (Moscovici, 1976/2008, 2000) and the concept of social identity (Duveen, 2001; Duveen & Lloyd, 1986; Howarth, 2002), we explore how experts’ by experience and registered nurses’ understandings of OST practices contain discourses about the identity of the participants (i.e., who they are, how they should be/behave) and the effects these may have on developing or hindering inclusive and bottom-up forms of patient and public involvement. Social representation theory enables us to study the relational and symbolic dimensions of participation (Campbell & Jovchelovitch, 2000), and thus can contribute to understanding citizenship as an “interactional matter” (Barnes et al., 2004) that is realized in the intersubjective space between clients and professionals in the health care.

Social representations are systems of social knowledge collectively constructed and reconstructed in communicative interaction and social practices with others (Moscovici, 1976/2008). In the case of developing OST services, it means that as common symbolic resources are shared by experts by experience on the one hand and registered nurses on the other hand to give meaning to their social and material worlds, and orient themselves within it, social representations inform the behaviours of these groups (Moscovici, 1984).

Identities become meaningful in social interactions and practices through processes of positioning the self in relation to social representations circulating in our environment; appropriating, reworking and/or contesting these representations (Duveen, 2001). The availability of different identity positions in these networks of meanings is framed and constrained by contextual norms and values (Duveen, 1993).

The relationship between how others represent the groups we belong to, and how we construct ourselves, becomes clear in the case of minority and socially excluded groups (e.g., Hodgetts et al., 2007; Howarth, 2002) as substance-abusing people who are vulnerable to specific kinds of epistemic injustice, such as testimonial injustice, stigmatization, and discrimination. In addition, substance-abusing peoples’ self-distrust in their experiences and knowledge as epistemic agents is a complex combination of shame, self-accusation, feelings of being oppressed, and a lack of proper concepts to express their own feelings (Auvinen et al., 2021).

In the case of developing OST services, experts’ by experience and registered nurses’ interactions in decision-making are asymmetric in terms of symbolic and material power (e.g., status, access to information), which may prevent involvees participating the meetings in ways that adequately reflect their own concerns and needs (also see Ansell & Gash, 2008; Barnes & Coelho, 2009).

## 3. Data and methods

The research data consists of recordings of meeting sessions held in one of the biggest cities in Finland between 2018 and 2019 in order to develop OST services both locally and regionally. The data analysed here include 18 two-hour meeting sessions conducted during the development project.

The meeting sessions were forums for the clients, experts by experience, and health care professionals to come together and share experiences, knowledge, and best practices related to OST. The meeting sessions were organized as part of a larger, EU-funded national development project aimed at supporting the social inclusion of clients with opioid addiction by strengthening their ability to function in the labour market.

There were in total 39 participants in the meeting sessions under scrutiny. In addition to clients and trained experts by experience, the participants included, for example, registered nurses, practical nurses, nursing students and other students, project workers, and various service managers. The professionals taking part in the meetings worked in public health centres, private clinics, and third-sector organizations. The number of participants varied between 8 and 28 across sessions, the most populated being the last one organized within the development project.

The meeting sessions under scrutiny were not the most optimal data for analysing the role of experts by experience as consultants and evaluators in the co-production of OST services. This is because, instead of mainly focusing on discussing the OST services, the meeting sessions were mostly concerned with the practical administrative issues, like the organization and scheduling of future meetings. In addition, the OST practices and policies were commented on and evaluated by only few experts by experience in the meeting sessions used as data in this study. The professionals participating in the meetings did not explicitly ask about experts’ by experience opinions and experience of OST services. Sharing one’s experiences and expressing one’s opinions therefore required initiative from the experts by experience themselves, which may not be an easy task in meetings full of professionals, not peers.

The topics of the meetings fell under two main themes—OST and the continuity of the meetings themselves after the completion of the development project. In terms of the former theme, the participants discussed, and sometimes argued about, various issues like the organization of client transfers between different service units, the service providers’ diverse attitudes towards the clients’ use of benzodiazepines (or “benzos”) during treatment and the role of experiential expertise and peer support in OST.

There was an issue that regularly aroused argumentation and controversy at the meetings, especially between the experts by experience and a group of registered nurses. The issue was that of drug screening, performed as part of the diagnosis of a client’s condition or as part of treatment monitoring. The analysis focuses on those cases where the experts by experience and a group of registered nurses argued about the practices of drug screening in the context of meeting talk.

In the analysis, we used two parallel analytic strategies to examine social representations and identities: (1) by using content analytical (Hsieh & Shannon, 2005) and (2) discourse analytical methods (Hall, 2001; Wetherell & Potter, 1992). The data analysis was initiated by using iterative thematic analysis to identify key themes (e.g., meanings, symbols) after which the text was reduced, grouped and designated as descriptive subcategories according to the content analysis. The subcategories were further combined as upper classes, of which the description of experts by experience consisted of them as actors in health care.

After limiting the data, discourse analysis was applied to analyse it. We examined the type of discourse through which the themes emerged in participants’ construction of social representations and identities (e.g., contestation, explanation or justiﬁcation). Through these strategies, we identified inter-relationship between the conversation themes including their sequential relationship within the conversation as well as the dynamics of the content within the conversation themes. In the course of repeated rounds of analysis, we developed a coding frame of themes and sub-themes and identiﬁed the discursive patterns through which they arose (See Renedo & Marston, 2011).

The focus of the discourse analysis was to look at how the meeting participants, experts by experience, and registered nurses used verbal statements and arguments to produce social representations, identity, and citizenship. The discourse analysis was carried out in three stages, of which the first was asked what kind of role for experts by experience as the developers of opioid substitution treatment is built in meeting sessions. The second phase of the analysis answered the question of how the meanings given by experts by experience to their position are related to the representations produced by nurses providing opioid substitution treatment. The third stage of the analysis answered the question of how the meanings made by experts by experience regarding treatment practices are related to a wider context of citizens’ access to the development of health care practices. Our data analysis is hermeneutic, whereby the subject of the study appears first as a certain kind, and understanding of it increases with different phases of analysis, continually opening up new perspectives on the data (Pietikäinen & Mäntynen, 2009, pp. 143–144).

## 4. Ethical considerations

The study has undertaken actions to gain ethics approval from the University Advisory Board on Ethics for studies and activities that involved gathering data from individual participants. The study has been conducted according to universal ethical principles (i.e., *Charter of Fundamental Rights of the European Union 2000*) and, in particular, according to *General Data Protection Regulation* (*Regulation EU 2016/679 of the European Parliament and of the Council on the protection of natural persons with regard to the processing of personal data and on the free movement of such data, and repealing Directive 95/46/EC*).

The study complied with applicable international, EU, and national legislations for the protection of personal data. The participants’ anonymity and data security have been protected. The participants of the meeting sessions were asked for their permission to record the meetings and to sign a written informed consent. The participants had sufficient information to give their informed consent. Personal data were handled by members of the research consortium who had been briefed about the security issues and the sensitive nature of the data. Dealing with all the needed information followed the ethical principles described in the study plans.

## 5. Results

We have explored the role of experiential knowledge in service co-production in order to develop OST services for high-risk opioid users by analysing (1) how the experts by experience attempt to legitimize their expertise that is based on experience in relation to the expertise of health care professionals that is based on professional abstract knowledge and (2) how the registered nurses justify their knowledge-based expertise in relation to the experiential knowledge of experts by experience.

Our analysis shows that experts by experience present themselves as experts of substance abuse problems whose experiential knowledge adds value to the professional knowledge of nurses in the rehabilitation of drug addicts. The health care professionals in turn primarily identify themselves as regulators who protect the correctness of their actions and show little interest in considering the experts’ by experience experiential knowledge of opioid addiction.

### 5.1. Identity of experts by experience as experts on living with addiction

Research data from meeting sessions show that the experts by experience attempt to legitimize their expertise based on experience by identifying themselves as experts of substance-abuse problems who are recovering from such problems. Only they can truly be said to “know” what policy and provision are like, rather than what its aims, intentions, and rationale are, since they have lived through these practices. Excerpt 1 reveals how experts by experience identify themselves as health care clients who have rid themselves of substance abuse. An expert by experience, EE1 demonstrates her rehabilitation by insisting that substitution treatment users be monitored more frequently. At the same time, she presents herself as a trusted client who is ready for a drug screening at any time:

*EE1:* *I think you take quite few drug tests at clients in opioid substitution treatment centres. I could go for tests more often because I want to show that I do not use drugs*. (Excerpt 1)

The expert by experience also differentiates herself from other OST patients by presenting them as untrustworthy people who have a tendency to trick the health care staff. In excerpt 2, an expert by experience (EE2) presents herself as a responsible actor in health care:

*EE2:* *But in fact, I am worried about the fact that many of the opiate substitution treatment patients may cheat in screenings*. (Excerpt 2)

By presenting herself as a responsible actor, the expert by experience points out that, she knows more about substance abuse and substance abuse clients than the health care staff do. The statement of expert by experience highlights what distinguishes service-user knowledge from professional expertise, that is, service-user knowledge is based on the client’s direct experience. The direct experience justifies the client’s status as an actor who is to be taken into account in patient recovery. What is distinctive in her perspective is that it rests on her role as the end user of policy and practice. Unlike health care professionals, the expert by experience “knows” through lived experience.

### 5.2. Identity of health care professionals as regulators

Health care professionals identify themselves as regulators whose duty is to carry out substitution treatment in accordance with professional knowledge by relying on (1) professional treatment practices, (2) professional ethics, and (3) their own experiential knowledge.

In excerpt 3, we can see how registered nurses (N1–N3) primarily identify themselves as regulators relying on professional treatment practices when they justify established OST to an expert by experience (EE3):

*EE3:* *In my opinion, there are quite few drug screenings taken at public health centres.*

*N1:* *Isn’t it so that, at about monthly intervals, it can take three weeks or it can be at weekly intervals, depending on the situation?*

*N2:* *According to the situation, yes.*

*N3:* *Yeah.*

*N2:* *Some need more frequent screening and some need a little bit less frequent screening, depending on the person’s treatment phase too.*

*N1:* *Hmm. Because this is rehabilitative substitution treatment*. (Excerpt 3)

When an expert by experience gives an opinion on the excessive time span of drug testing in health centres, the nurses imply that the expert by experience is wrong by invoking to professional treatment practices. When justifying OST practices, the nurses jointly produce an account of prevailing drug screening practices that are flexible on a case-by-case basis.

In their account, the nurses present the service system as a customer-oriented environment wherein it is the duty of the professionals to act in accordance with the values of the service system. By relying on professional treatment practices, as well as the values of health care, nurses jointly question the development proposal implied by an experienced expert.

In excerpt 4, a registered nurse (N4) identifies herself as a regulator by relying on professional ethics when she justifies established OST practices to an expert by experience (EE4):

*EE4:* *I have noticed and experienced that people with substance problems are good at manipulation, so even though you think you know the person, pretty harsh things can still be revealed about what the reality really is, and I have heard some nurse being laughed at because it can be cheated so easily.*

*N4:* *Our nurses do know—I believe that all nurses know—what you are saying.*

*EE4:* *Hmm.*

*N4:* *But our mission is to believe the client. I always say that we are on the clients’ side.*

*EE4:* *Yes, at least I work for my own sake. I mean I have recovered and stuff like that*. (Excerpt 4)

The expert by experience argues that the disadvantage of substitution treatment is that the nurses providing substitution treatment have too much trust in clients. Nurses do not know the behaviours of drug addicts because they do not share the same world with patients receiving substitution treatment. In her reply to the expert by experience, the registered nurse (N4) points out implicitly that according to her professional ethics she should rely on the patient. By appealing to professional ethics, she justifies her status as a professional actor having competence in patient care.

In excerpt 5, the registered nurse (N5) identifies herself as a regulator by relying on experiential knowledge when she justifies established OST practices to the expert by experience (EE5):

*EE5:* *I myself worry because there are so many other substitution treatment clients there; and how common cheating is, I mean, that the reality is something totally different than–*

*N5:* *We certainly get the exact information about what kind of pee there is. The lab will contact [us].*

*EE5:* *Hmm … Yes, but I just–*

*N5:* *I do know every trick of the trade too. [Laughing] I believe that I know.*

*EE5:* *Yes*. (Excerpt 5)

The expert by experience is concerned about whether nurses know that many clients receiving OST cheat the service system. She expresses her concern by subtly reflecting the commonness of cheating among clients receiving substitution treatment. She justifies her concern for legitimacy by presenting an account of the reality of the substance abuse world, which is different from that of others. The nurse (N5) interrupts the statement of expert by experience, pointing out that the expert by experience should not be worried about that nurses are unaware of the potential misconduct of those receiving substitution treatment. The nurse relies not only on the testing system (“the lab will contact” [us]) but also on her professional experience of client behaviour.

## 6. Discussion

As we have shown, the meeting sessions that potentially offer room for creativity and problem-solving fail to provide any new propositions for fixing the system. Experts by experience present themselves as experts of substance abuse problems whose experiential knowledge adds value to the professional knowledge of nurses in the rehabilitation of drug addicts. The health care professionals in turn primarily identify themselves as regulators who protect the correctness of their actions and show little interest in considering the experts’ by experience experiential knowledge of opioid addiction.

As apparent from the analysis, the experts by experience participate in the meetings as laymen, whose experience as service users legitimates their provision of knowledge from the perspective of users. Their expertise is based on individual experiences. It is not institutionalized, scholarly recognized, abstract, and conceptualized knowledge, justified by mediated, institution-based trust and power. However, the experts by experience have to cultivate their experiences with training, and with this process, they distance themselves from their individual experiences and the other service users and reframe their experiences as a source of knowledge to be applied for the purposes of professional practices and knowledge construction. With this process, they build a new identity in relation, first, to the professional system and practices, and second, to the other service users. Their subject position is in between the service user and the professional, but not identical with either of them.

By applying Fricker’s (2007) conceptualization of testimonial epistemic injustice, we argue that the professionals’ testimonial injustice towards experts by experience appears, shown by ignoring these former opioid users’ comments. The embodied knowledge of experts by experience is unattainable for professionals, so they suppress this unknown aspect in the body of professional knowledge. The suppression of the unknown as a form of institutional ignorance does not necessarily serve the interests of power but shows a commitment to the laws and ethics that protect vulnerable substance users.

### 6.1. Strength and limitations

Official statistics on practice in health care are based on quantitative data, which provides information in numbers about health care practices. However, for example, the number of experts by experience in planning and assessment groups does not illuminate the quality of their impact. Our research contributes to this shortage of knowledge using qualitative methodology, investigating interaction, and discourses, and is, thus, able to reveal the positions and roles of professionals and experts by experience in the co-production of services We have also been able to illuminate how different levels or scales of wilful ignorance in health institutions are not necessarily related to individual reasoning but to accountability in policy and regulation in OST.

The strength of this research lies in the methodological approach. Research based on quantitative data cannot reach the tensions in the interaction and identity negotiation. With the opportunity to study authentic interaction in the meetings of professional staff and experts by experience, we reveal the informal interaction and unequal relationships behind the official facade.

Our study focused on experts by experience without having detailed information about their background. A study by Renedo et al. (2015) reported the significance of educational and profession-specific background enhancing the position of experts by experience in the eyes of health care professionals. Thus, we recommend paying extra attention to the socio-economic position of experts by experience in the future, for it is related to problems of inequality.

The limitations of the research are related to the thin data on the social, economic, and cultural background of the experts by experience, which may strengthen or weaken their position in relation to the professionals. Previous studies have paid attention to the importance of the resources originating from their middle-class background, as these resources may help the experts by experience to adapt to healthcare management structures and processes and leave the less advantaged experts by experience powerless (Renedo et al., 2015).

Conclusions regarding the significance of socio-economic background may have implications for expertise by experience in the co-production of health care services. Experts by experience with a low social position are taken less seriously as “knowers” by the professionals, but taking into account their poorer resources for coping with rehabilitation, they should be listened to even more carefully for that reason.

## 7. Conclusions

Health care services are highly regulated by a variety of laws. One of the most prominent is the law regulating the competencies of professional personnel, for it provides the basis for expertise and the ethical code for treating vulnerable human beings. The health care professions have clearly defined territories with strict boundaries, relative independent knowledge, and training institutions. The professional system, with a certain knowledge basis and related institutions and practices, is based on distancing itself from lay knowledge and practices. Professional legitimation is based on this abstracted knowledge and the epistemology of knowing.

Though the relations of different professional groups in health care institutions are strictly hierarchical, professional legitimation is applied to every group. Although the nurses claim legitimation with a special quality of work—in other words, care—they lean on the dominating principle of the profession, on medicine (Popay & Williams, 1996; Thompson et al., 2012; Weiner, 2009).

Despite the dominating position of professions in health care, in recent legislation, the erasing of special needs and relations in society has to be considered in regulating and running health care services. The participation of patients has been one of the prominent reforms implemented in health care. The goal of their involvement or of client-centred thinking is often emphasized; however, the implementation is not simply due to the strongly institutionalized knowledge and related working patterns and practices in health care (Popay & Williams, 1996; Thompson et al., 2012; Weiner, 2009).

With the method of promoting the participation of citizens, clients, and patients in the planning and development of health care services, the effectiveness of treatment is assumed to be increased and the ethics of treatment carried out. The participation of patients is promoted by policy organizations at international and national levels. The EU is engaged in this goal with the European Patients’ Forum, which is a higher-level organization that represents patients’ organizations in the member states of the EU (European Patient Forum, 2018). The strategy of the World Health Organization emphasizes the participation of patients in order to decrease the substantial inequality of health among populations (Boyce & Brown, 2017; WHO, 2013). At the national level, Finland has adjusted the legislation in a way that creates grounds for adopting this method or practice in planning and implementing health care services (among other legislation: Constitution 17.6.731/1999, Law of Health Care 31.12.2010/1326). In other words, promoting the participation of patients and expertise by experience is of primary importance and cannot be ignored in practices and relations in health care services.

## Note

1. The concept of experiential knowledge refers to Borkman’s (1976) definition. The concept is cognate to Michael Polanyi’s personal knowledge, as well as to Bertrand Russell’s contrast of Knowledge by Acquaintance and by Description. Experiential knowledge is grounded in lived experiences, in particular, adversities in life that are difficult for outsiders to comprehend without similar type of experiences. Experiential knowledge is not generated automatically but requires some training, practice, or reflective processing.
